# Built Environment and SARS-CoV-2 Transmission in Long-Term Care Facilities: Cross-Sectional Survey and Data Linkage

**DOI:** 10.1016/j.jamda.2023.10.027

**Published:** 2024-02

**Authors:** Maria Krutikov, Oliver Stirrup, Chris Fuller, Natalie Adams, Borscha Azmi, Aidan Irwin-Singer, Niyathi Sethu, Andrew Hayward, Hector Altamirano, Andrew Copas, Laura Shallcross

**Affiliations:** a Institute of Health Informatics, University College London, London, UK; b Institute for Global Health, University College London, London, UK; c Surveillance Testing and Immunity, UK Health Security Agency, London, UK; d Institute for Environmental Design and Engineering, University College London, London, UK; e Institute of Epidemiology and Health Care, University College London, London, UK

**Keywords:** COVID-19, long-term care, built environment, infection prevention & control, infection transmission, older adults

## Abstract

**Objectives:**

To describe the built environment in long-term care facilities (LTCF) and its association with introduction and transmission of SARS-CoV-2 infection.

**Design:**

Cross-sectional survey with linkage to routine surveillance data.

**Setting and Participants:**

LTCFs in England caring for adults ≥65 years old, participating in the VIVALDI study (ISRCTN14447421) were eligible. Data were included from residents and staff.

**Methods:**

Cross-sectional survey of the LTCF built environment with linkage to routinely collected asymptomatic and symptomatic SARS-CoV-2 testing and vaccination data between September 1, 2020, and March 31, 2022. We used individual and LTCF level Poisson and Negative Binomial regression models to identify risk factors for 4 outcomes: incidence rate of resident infections and outbreaks, outbreak size, and duration. We considered interactions with variant transmissibility (pre vs post Omicron dominance).

**Results:**

A total of 134 of 151 (88.7%) LTCFs participated in the survey, contributing data for 13,010 residents and 17,766 staff. After adjustment and stratification, outbreak incidence (measuring infection introduction) was only associated with SARS-CoV-2 incidence in the community [incidence rate ratio (IRR) for high vs low incidence, 2.84; 95% CI, 1.85–4.36]. Characteristics of the built environment were associated with transmission outcomes and differed by variant transmissibility. For resident infection incidence, factors included number of storeys (0.64; 0.43–0.97) and bedrooms (1.04; 1.02–1.06), and purpose-built vs converted buildings (1.99; 1.08–3.69). Air quality was associated with outbreak size (dry vs just right 1.46; 1.00–2.13). Funding model (0.99; 0.99–1.00), crowding (0.98; 0.96–0.99), and bedroom temperature (1.15; 1.01–1.32) were associated with outbreak duration.

**Conclusions and Implications:**

We describe previously undocumented diversity in LTCF built environments. LTCFs have limited opportunities to prevent SARS-CoV-2 introduction, which was only driven by community incidence. However, adjusting the built environment, for example by isolating infected residents or improving airflow, may reduce transmission, although data quality was limited by subjectivity. Identifying LTCF built environment modifications that prevent infection transmission should be a research priority.

The COVID-19 pandemic's impact in long-term care facilities (LTCFs) has highlighted substantial gaps in knowledge around infection prevention in care settings. Approximately 390,000 people live in 11,000 LTCFs for older adults in England and they are especially vulnerable to severe outcomes from COVID-19 because of advanced age, frailty, and comorbidities.^1^ In the first pandemic wave, LTCF residents experienced a 30-fold increase in COVID-19 mortality risk compared with age-matched adults in private dwellings.^2^ Further repercussions of infection outbreaks in LTCFs include emotional distress for residents and relatives from restricted visiting,^3^ negative impacts on care, financial losses from closures, and reputational damage. LTCFs implemented a package of COVID-19 control measures to protect staff and residents, but the simultaneous introduction of multiple interventions has limited the generation of evidence to support their use.^4^^,^^5^

In England, LTCFs for older people deliver a mix of residential, nursing, and dementia care. Most care is provided by the independent sector, consisting of for-profit and not-for-profit organizations; local authorities (LAs) provide the remainder.^6^ Built environments (the human-made structures where people live and work) vary, but to date large-scale studies have not captured this diversity as they could access limited relevant variables from administrative datasets. SARS-CoV-2 predominantly spreads through respiratory droplets or airborne aerosols. Transmission is therefore greater within crowded, poorly ventilated spaces with lower humidity.^7^, ^8^, ^9^ Within LTCFs, crowding is a risk factor for SARS-CoV-2.^10^ However, many potentially important factors have not been explored, including the influence of ventilation, air quality and temperature, number of storeys, and whether buildings have been repurposed, despite evidence from other settings.^11^^,^^12^

Infection prevention strategies within LTCFs include entry regulation, such as restricting visitors, contact regulation using personal protective equipment, surveillance, and outbreak control measures such as cohorting, where infected residents are isolated together.^5^ However, negative consequences, including social isolation and depression, are well-documented^12^^,^^13^ and recommendations for environmental adaptations that may be better tolerated are sparse.^14^

To test the hypothesis that built environments vary significantly among LTCFs and that this variation is associated with SARS-CoV-2 infection in LTCFs, our objectives were to describe the variation in built environment and identify factors associated with SARS-CoV-2 introduction and transmission. We designed detailed surveys to collect data on unexplored features of the built environment that we linked to infection screening data.

## Methods

Between April 4 and November 2, 2021, we performed a cross-sectional survey about the built environment in LTCFs for older adults (≥65 years) in England participating in the VIVALDI study (ISRCTN14447421).^15^ Questionnaires were linked to routine data from staff and residents on asymptomatic and symptomatic SARS-CoV-2 testing and vaccinations between September 1, 2020, and March 31, 2022. Study design and reporting follow the CROSS^16^ and RECORD checklists.^17^

### Procedures

Survey design was led by an infectious diseases clinician (M.K.) and building scientist (H.A.) with oversight from a public health expert (L.S.). It comprised 19 questions with multiple-choice and free-text answers pertaining to size, crowding, and airflow (ventilation, air quality, temperature), based on literature and experience. To minimize time pressures, we collected information that was relatively accessible including subjective assessments (survey provided in Supplementary Material, Section 2). Piloting was conducted with 2 LTCF managers whose feedback clarified wording.

Using a convenience sample, LTCFs were approached by project managers from 2 for-profit and 2 not-for-profit providers. Questionnaires were distributed electronically and completed, once per LTCF, by maintenance or management staff. Personal identifiers were not collected or stored. Providers consented to aggregate data collection on enrollment to VIVALDI.^15^ Incentives were not offered and reminders were sent until November 1, 2021. Responses were stored in the institutional secure data repository.^18^

**Fig. 1:**
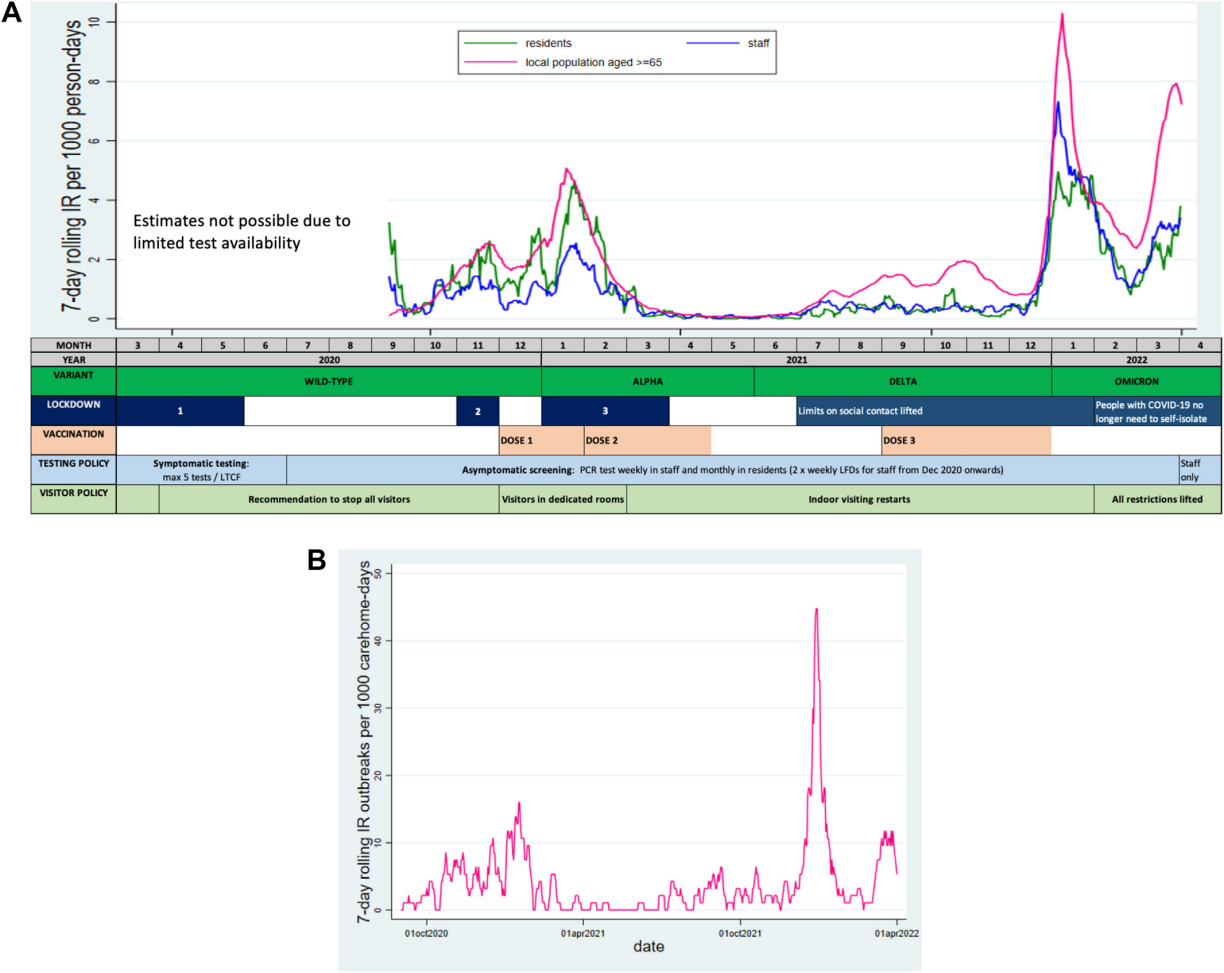
(A) Seven-day rolling incidence rate of SARS-CoV-2 infection among staff and residents compared with local SARS-CoV-2 incidence, with timeline of national social care SARS-CoV-2 prevention policies in England. Comparison is made with SARS-CoV-2 incidence rate among adults >65 years in the local community based on national data. Policy timeline summarizes key changes over the pandemic period up to the end of the study period including dominant variant, national lockdowns, vaccination rounds, testing policy, and visiting policy. (B) Seven-day rolling incidence rate of SARS-CoV-2 outbreaks in participating LTCFs.

The analysis period was chosen based on the COVID-19 screening program in England (Figure 1A), as this enabled identification of study participants. Regular asymptomatic polymerase chain reaction (PCR) testing was fully established in LTCFs from September 2020 to April 2022 (weekly in staff, monthly in residents). From December 2020, additional testing using lateral flow devices (LFDs) was introduced.^19^ Using LFD/PCR test results, participants were linked to their care home's CQC-ID, a unique number allocated by the Care Quality Commission (CQC). A person-level pseudo-identifier, based on National Health Service (NHS) number, allowed linkage to datasets on vaccination and nucleocapsid-antibody results (acquired from SARS-CoV-2 infection, collected as part of VIVALDI^20^). Linkage using CQC-ID to Capacity Tracker, a self-completed tool for tracking LTCF capacity,^21^ provided bed occupancy and staffing data. Providers directly supplied data on bed funding. Linkage to national datasets on SARS-CoV-2 incidence and deprivation used LTCF postcode. Further linkage details are described elsewhere^20^ (Supplementary Material).

Staff and residents were included if they had a valid pseudo-identifier that could be linked by at least one PCR/LFD test within the analysis period to an LTCF that had completed the survey. Staff or resident status was defined using published methods.^20^

### Outcomes and Covariates

Two primary outcomes were included describing infection introduction and transmission: (1) incidence of SARS-CoV-2 infection in residents (both introduction and transmission); and (2) incidence of SARS-CoV-2 outbreaks (introduction). Secondary outcomes describing infection transmission were outbreak size (including both staff and residents) and outbreak duration, defined by days between the first and last positive test. These outcomes provide insight into how infection spreads after LTCF entry and may therefore better identify susceptible facilities.

Cases were defined by positive LFD/PCR and only tests >90 days apart from 1 individual were included.^22^^,^^23^ As per national guidance,^24^ outbreaks were defined by at least 2 PCR/LFD-confirmed cases in an LTCF within 14 days and continued until there were no new cases over 28 days. This definition was modified to include at least 1 resident case, as these infections were probably acquired in the LTCF, whereas infections in staff may have been community-acquired. Outbreaks were included if the first day preceded the study end.

A “base” time-varying model was built using factors with known associations with study outcomes. To preserve sample size, we did not model building factors jointly, as questionnaires varied in completeness. Instead, separate models to estimate the relationship between each building factor and outcome were fitted, adjusting for the “base” model. Confounders in “base” models included person-level factors: sex, age, prior SARS-CoV-2 infection (defined by a previous positive PCR/LFD and/or nucleocapsid-antibody), and vaccination; facility-level factors: bed number, occupancy, total staff, ownership, bed funding; and local factors: socioeconomic deprivation level^25^ and local SARS-CoV-2 incidence,^26^ Supplementary Table 1. Where these varied over time, a monthly average was used.

Survey building factors were included if responses were ≥80% complete and ≤90% of answers were the same. As accuracy could not be verified, temperatures above 30°Celsius were considered missing.

### Statistical Analysis

We modeled the 7-day rolling incidence rate of SARS-CoV-2 infections and outbreaks among staff and residents. Participants were considered at-risk between the dates of their first and final PCR/LFD test within participating LTCFs. Participants with final tests in the study's last 3 months remained at-risk until the study end, to account for missed tests. Following a positive test, individuals were removed for 90 days.

To estimate risk factors for SARS-CoV-2 incidence among residents, multivariable Poisson regression models were built using participant-level data and individual- and facility-level frailty terms to account for clustering, with monthly number of person-level at-risk days as the exposure term. This approach was replicated for outbreak incidence using facility-level data, with only a facility-level frailty term and the monthly number of facility-level at-risk days as the exposure term.

Risk factors for secondary outcomes of outbreak size, and duration, were modeled using multivariable negative binomial regression with facility-level frailty terms. As minimum outbreak size was 2 and outbreak duration was 1, these values were subtracted from these outcomes before analysis.

Analyses were conducted at the person-level for infection incidence and facility-level for outbreak-related outcomes. All models were adjusted for calendar month. For continuous variables, linearity of association was assessed using likelihood-ratio tests (LRTs) comparing model fit between linear and polynomial factors. In “base” models, polynomials were retained for nonlinearly associated covariates. To facilitate interpretation of results, building factor polynomials were not retained; nonlinearly associated continuous covariates were instead categorized into terciles.

To explore impact of the Omicron variant,^27^^,^^28^ we created an indicator for the Omicron-dominant period (after December 1, 2021)^29^ and assessed interactions with variables describing immunity, and building factors, using LRT. Significant interaction terms were retained in the “base” model. Full stratification of analyses was considered when multiple interactions linked to Omicron dominance were identified.

Analyses were conducted using Stata v17.0.

### Ethical Approval

This study was granted ethical approval by the South-Central Hampshire B NHS Research Ethics Committee (ref:20/SC/0238). Legal basis for data linkage is provided by the COVID-19: notice under regulation 3(4) of the Health Service (Control of Patient Information) Regulations 2002.^30^

## Results

### Description of Building Environment

**Supplementary Fig. 1:**
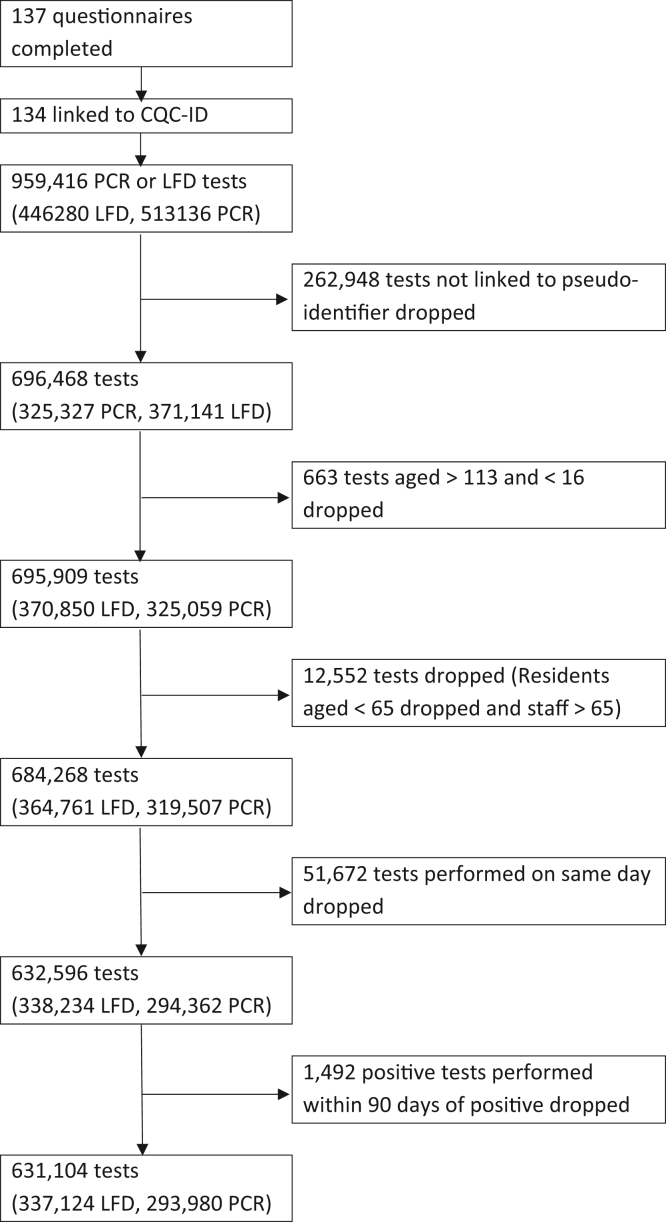
Inclusion flow diagram.

Of 151 questionnaires, 137 were completed and 134 (88.7%) could be linked to a CQC-ID (Supplementary Figure 1). Of these, 105 (78.4%) were completed by a manager, 19 (14.2%) by the maintenance officer, and the rest unknown. Where stated, almost half were completed in April 2021 (56 of 119), and the remaining 63 between May and November (1 in November). LTCFs were distributed across England and most (116 of 134, 86.6%) were for-profit.

Completeness varied by question, from 6% (8 of 134) to 97% (130 of 134). Of 128 LTCFs, 104 (81.2%) were purpose-built and the remainder had been converted. At least half of LTCFs had 2 storeys and the median bedroom number was 52 (IQR 41–65). One LTCF reported shared bedrooms; however, in 22.5% (27 of 134) staff and residents shared bathrooms (Table 1).

**Table 1:** Building Survey Responses and Proportion of Questions That Were Completed

Building Factor	No. Completed (%) n = 134	Summary of Responses
Number of rooms (Mean, SD)		
Bedrooms	123 (91.8)	54.65 (21.40)
Common rooms	128 (95.6)	3.97 (2.60)
Dining rooms	130 (97.0)	2.33 (1.20)
Kitchens	129 (96.3)	1.53 (1.10)
Toilets	125 (93.3)	9.34 (6.77)
Staircases	130 (97.0)	3.37 (2.05)
Corridors	129 (96.3)	6.39 (4.02)
Storeys	111 (82.8)	2.21 (0.56)
Building type	128 (95.5)	
Purpose-built		104 (81.2%)
Converted		24 (18.8%)
Presence of shared bedrooms (% responses)	126 (94.0)	10 (8.0%)
Number of shared bathrooms (between residents) (mean, SD)	102 (76.1)	1.5 (1.07)
Presence of shared toilets (staff and residents) (% responses)	120 (89.6)	27 (22.5%)
Air temperature (°Celsius) (mean, SD)		
Dining room	38 (28.4)	22.77 (2.66)
Common room	52 (38.8)	22.87 (2.45)
Bedroom	32 (23.9)	22.59 (2.86)
Perceived air quality (common room) (% responses)	115 (85.8)	
Too humid		5 (4.3%)
Humid		9 (7.8%)
Slightly humid		16 (13.9%)
Just right		70 (60.9%)
Slightly dry		10 (8.7%)
Dry		3 (2.6%)
Too dry		2 (1.7%)
Perceived air quality (dining room) (% responses)	115 (85.8)	
Too humid		6 (5.2%)
Humid		7 (6.1%)
Slightly humid		18 (15.7%)
Just right		75 (65.2%)
Slightly dry		6 (5.2%)
Dry		2 (1.7%)
Too dry		1 (0.9%)
Perceived air quality (bedroom) (% responses)	113 (84.3)	
Too humid		4 (3.5%)
Humid		7 (6.2%)
Slightly humid		10 (8.7%)
Just right		82 (72.6%)
Slightly dry		6 (5.3%)
Dry		3 (2.7%)
Too dry		1 (0.9%)
Cleaning frequency–vacuuming (% responses)	111 (82.8)	
Daily		108 (97.3%)
Several times a week		2 (1.8%)
Weekly		1 (0.9%)
Several times a month		0 (0)
Monthly		0 (0)
Cleaning frequency–washing floor (% responses)	108 (80.6)	
Daily		91 (84.3%)
Several times a week		8 (7.4%)
Weekly		7 (6.5%)
Several times a month		1 (0.9%)
Monthly		1 (0.9%)
Cleaning frequency—sweeping (% responses)	105 (78.4)	
Daily		103 (98.1%)
Several times a week		1 (1.0%)
Weekly		1 (1.0%)
Several times a month		0 (0)
Monthly		0 (0)
Ventilation type–dining room (% responses)	54 (40.3)	
Central air conditioning		29 (53.7%)
Cassette ceiling unit		2 (3.7%)
Portable unit exhaust pipe		1 (1.9%)
Mechanical extraction unit		9 (16.7%)
Freestanding		9 (16.4%)
Unknown		4 (7.4%)
Ventilation type–common room (% responses)	67 (50.0)	
Central air conditioning		8 (11.9%)
Cassette ceiling unit		9 (13.4%)
Portable unit exhaust pipe		2 (3.0%)
Mechanical extraction unit		8 (11.9%)
Freestanding		35 (52.2%)
Unknown		5 (7.5%)
Ventilation type–bedroom (% responses)	52 (38.8)	
Central air conditioning		32 (61.5%)
Cassette ceiling unit		3 (5.8%)
Portable unit exhaust pipe		0 (0)
Mechanical extraction unit		9 (17.3%)
Freestanding		4 (7**.7**%)
Unknown		4 (7.7%)
Heating–dining room (% responses)	128 (95.6)	
Central heating		127 (99.2%)
Other		1 (0.8%)
Heating–common room (% responses)	124 (92.6)	
Central heating		123 (99.2%)
Other		1 (0.8%)
Heating–bedroom (% responses)	109 (81.3)	
Central heating		108 (99.1%)
Other		1 (0.9%)
Presence of humidifiers/air purifiers–dining room (% responses)	20 (14.9)	2 (10.0%)
Presence of humidifiers/air purifiers—bedroom (% responses)	15 (11.2)	3 (20.0%)
Presence of condensation (% responses)	124 (92.6)	12 (9.7%)
Presence of outdoor space (% responses)	124 (92.6)	121 (97.6%)
Maximum people in dining room at one time (mean, SD)	94 (70.2)	13.71 (7.49)
Maximum people in common room at one time (mean, SD)	101 (75.4)	12.02 (8.16)

**Supplementary Fig. 2:**
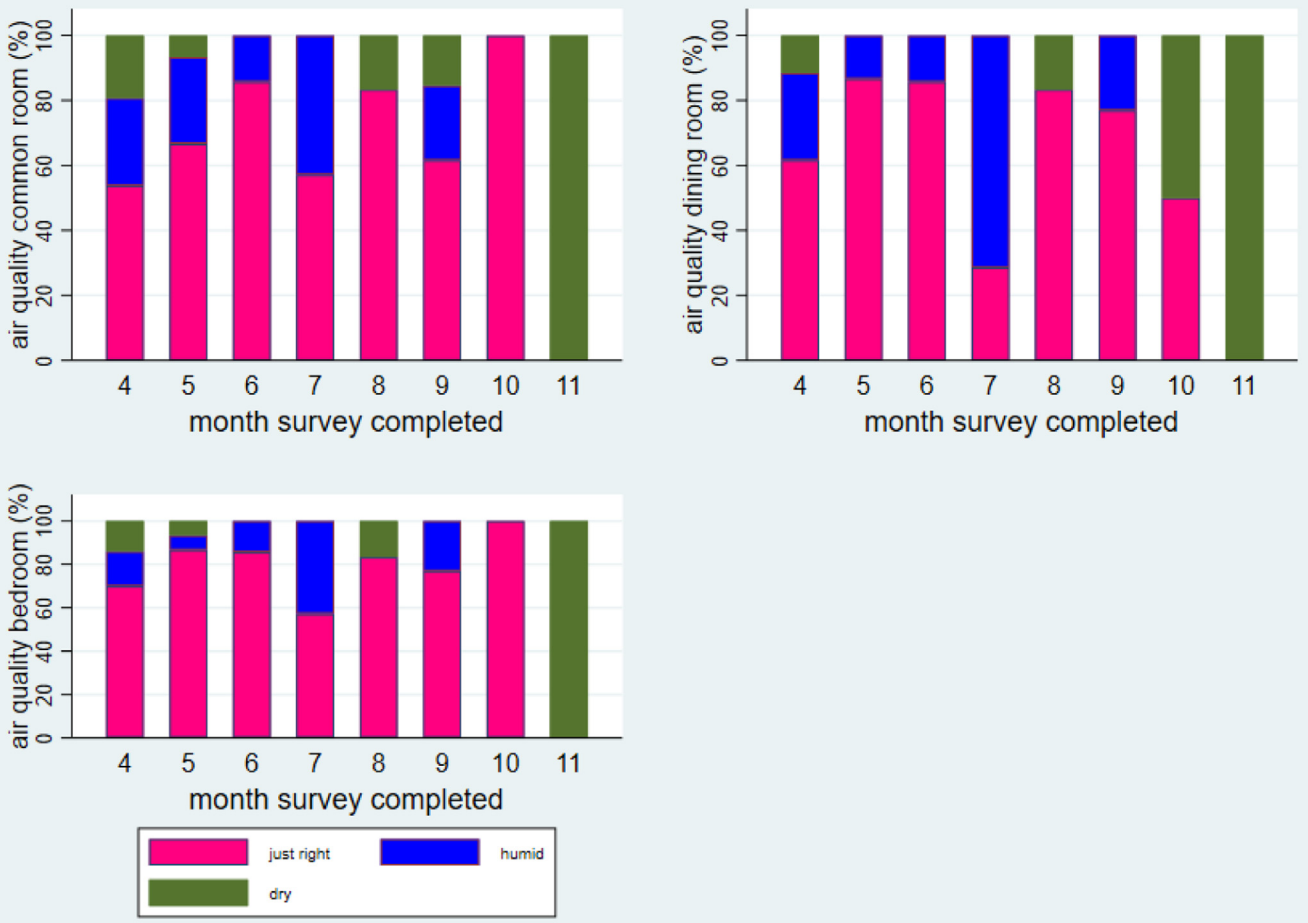
Perceived air quality reported by month of survey completion in common room, dining room, and bedroom.

Most were cleaned every day and perceived air quality as “just right” instead of “dry” or “humid.” Air quality and temperature did not vary by survey month (Supplementary Figure 2, Supplementary Table 2). Almost one-tenth reported condensation (12 of 124, 9.7%), most had outdoor space (121 of 124, 97.6%), and almost all used central heating (108 of 109, 99.1%). More than half reported ventilation type: central air conditioning was most common in dining rooms (29 of 54, 53.7%) and bedrooms (32 of 52, 61.5%), whereas freestanding fans predominated in common rooms (35 of 67, 52.2%) (Table 1).

### Description of Cohort

**Supplementary Fig. 3:**
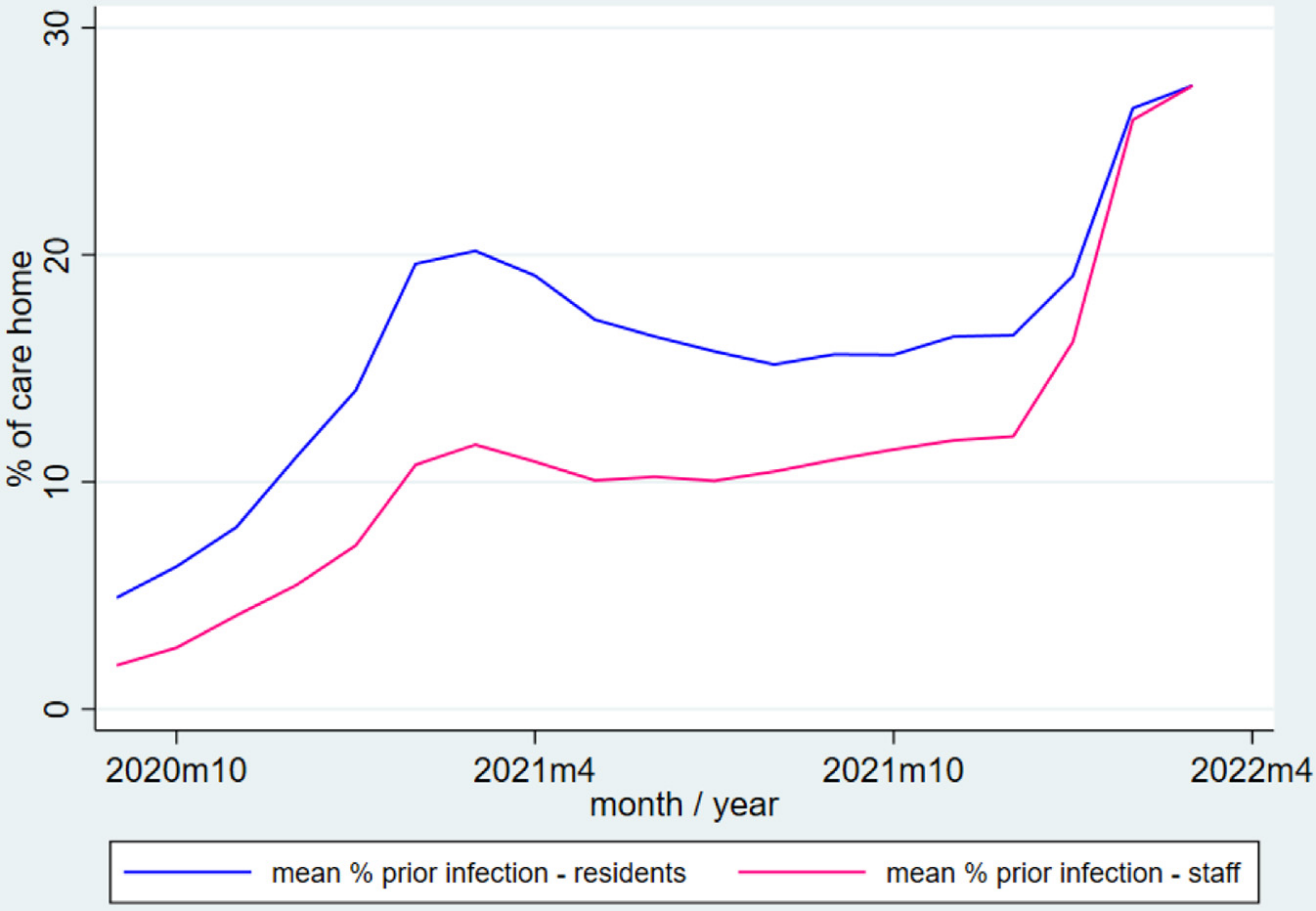
Mean proportion of LTCF with evidence of prior infection, by staff and residents (September 1, 2020–March 31, 2022).

**Supplementary Fig. 4:**
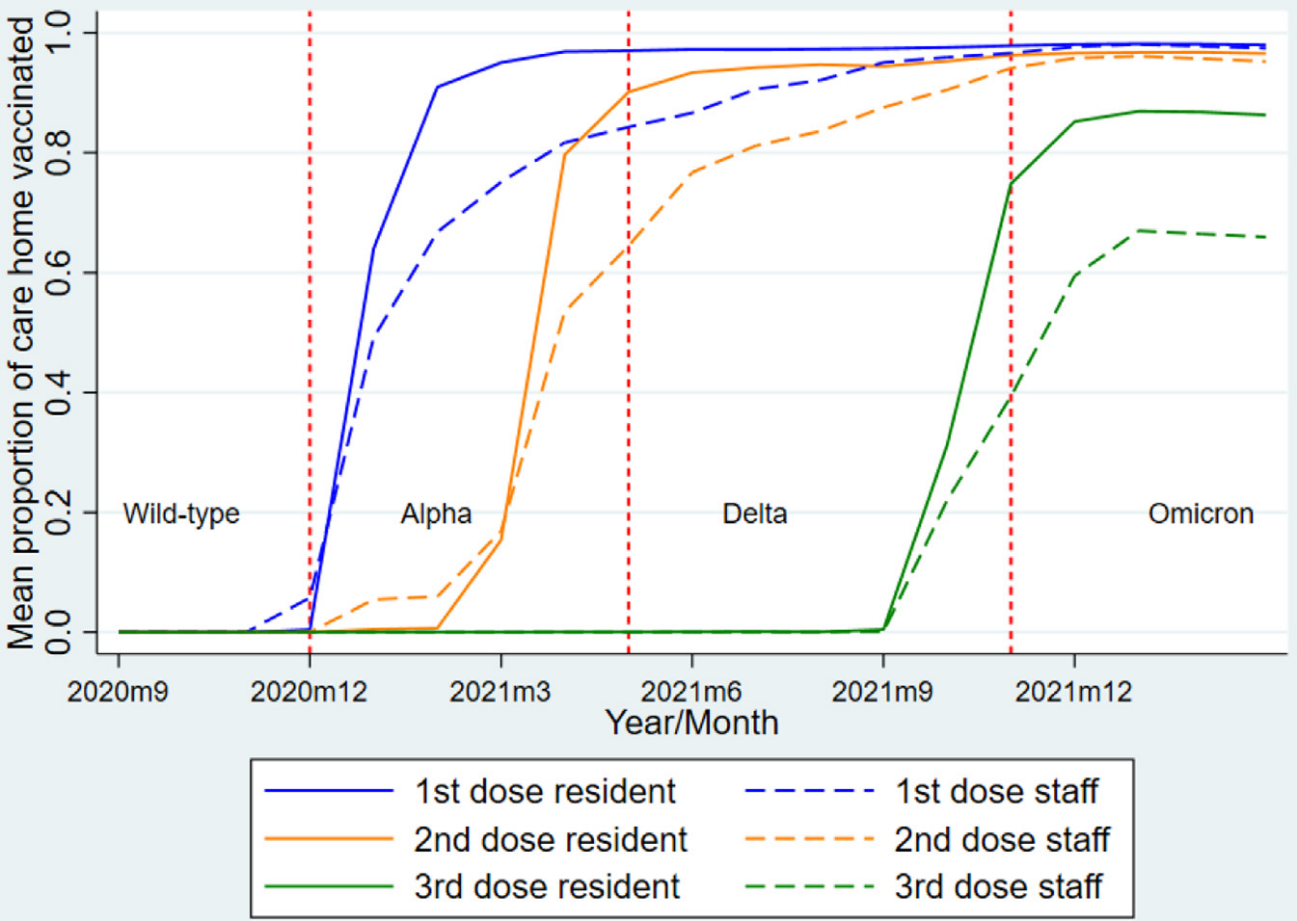
Mean proportion of the LTCFs that received 1, 2, and 3 vaccination doses, by staff and resident (September 1, 2020–March 31, 2022). Wild-type interval September 1, 2020–December 31, 2020; Alpha interval January 1, 2021–May 31, 2021; Delta interval June 1, 2021–November 30, 2021; Omicron interval December 1, 2021–March 31, 2022.

Data on infection and related outcomes were available for 13,010 residents and 17,766 staff (Supplementary Figure 1). Overall, 21,140 of 30,776 (68.7%) were female and median age was 47 (IQR 33.6–56.9) in staff and 83.5 (74.6–90.0) in residents (Table 2). Median follow-up was 104 days (9–334) per participant, comparable between staff and residents (102 vs 106 days). Per LTCF, the median number of staff was 48 (32–68) and beds was 51 (42–66), 73.8% (52.7%–85.7%) of which were LA-funded and 22.9% (0.0%–50.0%) were funded for dementia care (Table 3). Vaccination coverage and infection exposure increased over time (Supplementary Figures 3 and 4).

**Table 2:** Baseline Demographics: Person Level

Baseline Demographics	Number (%)
Number participants	30,774
Staff	17,766 (57.7)
Residents	13,008 (42.3)
Sex	
Male	9567 (31.1)
Female	21,140 (68.7)
Unknown	68 (0.2)
Age (median, IQR, range)	60 (43–80.6, 16–110.8)
Staff	47 (33.6–56.9, 16–65)
Residents	83.5 (74.6–90, 64–110.8)

**Table 3:** Baseline Demographics: Facility Level

Baseline Demographics	Number (%) Median (IQR, Range)
Number of LTCFs	134
Regions	
London	11 (8.2)
South-East	17 (12.6)
East of England	11 (8.2)
South-West	14 (10.4)
North-West	20 (14.8)
North-East	17 (12.6)
East Midlands	23 (17.0)
West Midlands	11 (8.2)
Yorkshire and Humber	11 (8.2)
IMD index	5 (3–8, 1–10)
LTCF type	
For-profit	116 (86.6)
Not-for-profit	18 (13.4)
Total staff∗	48 (32–68, 0–189)
Total beds∗	50.5 (42–66, 7.3–123)
Staff:resident ratio∗	0.8 (0.7–1.0, 0.3–2.6)
Bed:resident ratio∗	1.2 (1.1–1.4, 1–4.9)
Proportion LA-funded beds∗	73.8 (52.7–85.7, 0–100)
Proportion dementia beds∗	22.9 (0–50, 0–100)
Staff dose 2 vaccination coverage∗ (%)	75.6 (0–92.9, 0–100)
Resident dose 2 vaccination coverage∗ (%)	88.4 (0–96.4, 0–100)
Proportion staff with prior infection∗ (%)	7.9 (0–17.4, 0–100)
Proportion residents with prior infection∗ (%)	11.1 (3.3–24.4, 0–100)

IMD index, Index of Multiple Deprivation–ranges from 1 to 10, 1 is most deprived and 10 is least.

∗ Adjusted for person level: age, prior infection, receipt of second vaccine, sex; facility-level: Index of Multiple Deprivation, local SARS-CoV-2 incidence rate, for-profit status, number of beds, number of staff, number of residents, bed-to-resident ratio, resident-to-staff ratio, proportion residents with prior infection, proportion staff with prior infection, proportion staff vaccinated, proportion residents vaccinated.

**Supplementary Fig. 5:**
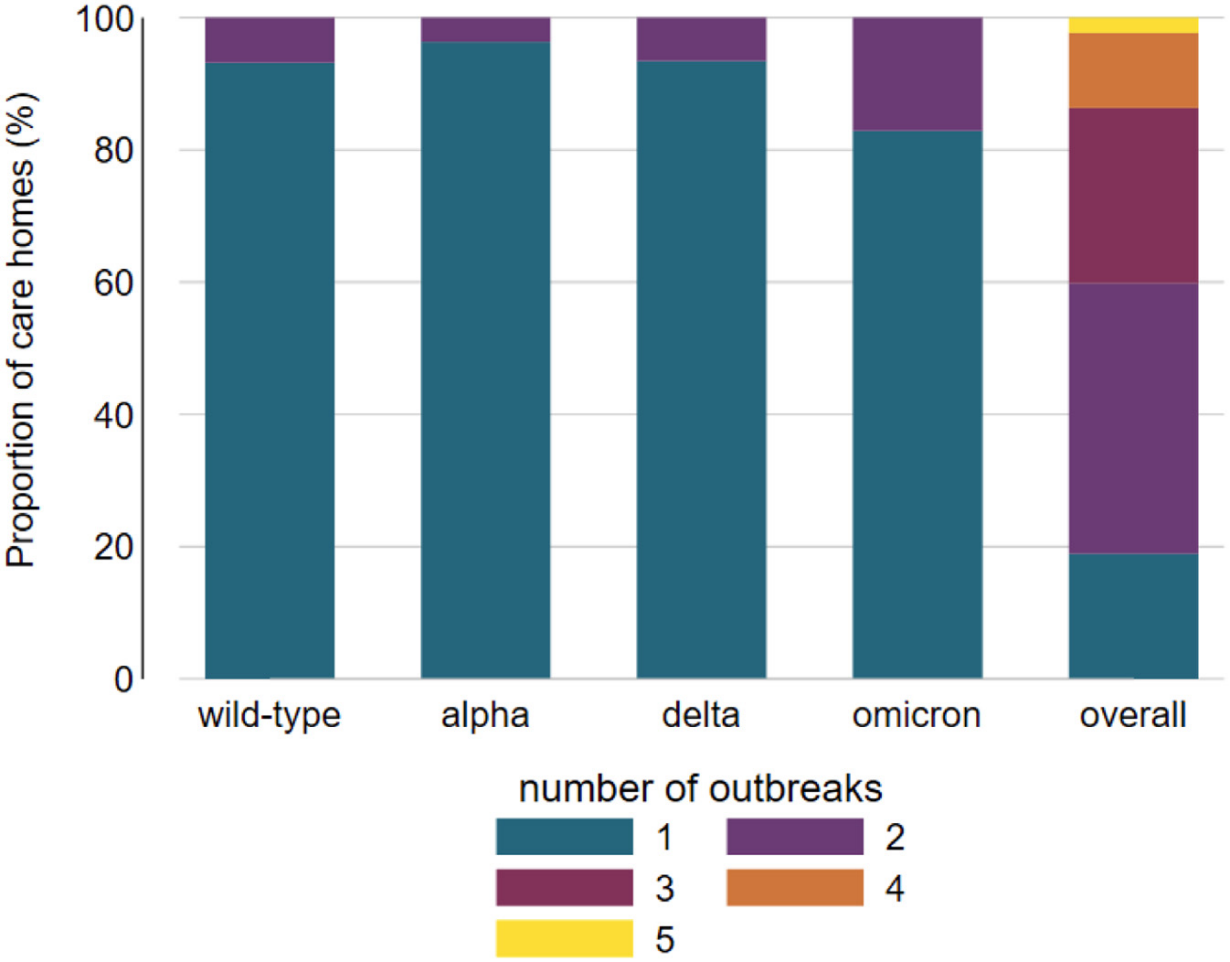
Number of outbreaks per long-term care facility by dominant variant and overall. Wild-type interval September 1, 2020–December 31, 2020; Alpha interval January 1, 2021–May 31, 2021; Delta interval June 1, 2021–November 30, 2021; Omicron interval December 1, 2021–March 31, 2022.

Seven-day rolling incidence rates of infection and outbreaks in residents followed similar trends to staff and reflected national epidemiology. Peaks occurred with Alpha variant dominance (October 2020–March 2021) and Omicron emergence (January–April 2022) (Figure 1A and B). Overall, 313 outbreaks occurred, with a median of 2 per LTCF (IQR 2–3). Characteristics varied over time with greatest outbreak number, size, and duration during Omicron dominance (Supplementary Figure 5, Supplementary Table 3).

## Risk Factors for Introduction and Transmission of Infection

“Base” models are presented in Supplementary Table 4, Supplementary Table 5, Supplementary Table 6, Supplementary Table 7. Significant associations with building characteristics are summarized. For categorical variables with *P* < .05, factors differing from the reference category are described. Factors excluded because of low response rate or variability were shared bedrooms, vacuuming and sweeping frequency, heating, humidifiers, condensation, and outdoor space.

For the first primary outcome of incidence of resident infections, 14 of 22 building factors had an interaction with the Omicron period. We therefore also stratified by Omicron dominance. Overall, additional storeys reduced infection rate by 36% [adjusted incidence rate ratio (aIRR), 0.64 per storey; 95% CI, 0.43–0.97; *P* = .036]. Factors associated with greater infection rate were purpose-built vs converted buildings (1.99; 1.08–3.69; *P* = .028), and those with more bedrooms (1.04 per bedroom; 1.08–3.69; *P* < .001) (Table 4).

**Table 4:** Mixed Effects Adjusted Person-level Poisson Regression Models of Incidence of Infection in a Resident,∗ Overall and Stratified by Pre-Omicron and Omicron-dominant Periods

Building Factors	Unstratified				Stratified – Pre-Omicron				Stratified – Omicron			
	aIRR	*P* Value	95% CI		aIRR	*P* Value	95% CI		aIRR	*P* Value	95% CI	
No. storeys	0.64	.036	0.43	0.97	0.51	.030	0.28	0.94	0.85	.56	0.50	1.45
Purpose built vs converted	1.99	.028	1.08	3.69	1.06	.90	0.46	2.42	2.92	.006	1.36	6.25
No. bedrooms†	1.04	<.001	1.02	1.06	1.04	.006	1.01	1.07	1.02	.08	1.00	1.05
No. common rooms†	1.01	.83	0.91	1.12	1.04	.59	0.90	1.20	0.98	.79	0.86	1.12
No. dining rooms†	1.09	.48	0.87	1.36	1.04	.79	0.77	1.40	0.98	.87	0.73	1.31
Presence of shared bathrooms (staff with residents)	0.75	.30	0.43	1.30	0.55	.12	0.25	1.17	0.96	.92	0.48	1.95
Presence of shared bathrooms (between residents)	1.23	.73	0.38	3.97	1.51	.61	0.32	7.17	1.28	.74	0.30	5.39
Ventilation–common room												
Freestanding fan	Ref	.56			Ref	.97			Ref	.42		
Cassette ceiling unit	0.84	—	0.33	2.11	0.79	—	0.24	2.57	1.01	—	0.23	4.46
Portable unit exhaust pipe	1.00	—	0.17	5.97	1.04	—	0.12	9.32	1.20	—	0.07	21.34
Mechanical extract units	1.99	—	0.78	5.07	1.15	—	0.37	3.62	6.37	—	1.14	35.52
Central air conditioning	1.67	—	0.58	4.79	0.95	—	0.25	3.57	0.80	—	0.14	4.60
Unknown	0.69	—	0.21	2.20	0.55	—	0.13	2.39	1.16	—	0.18	7.57
Ventilation–dining room												
Central air conditioning	Ref	.08			Ref	.037			Ref	.68		
Cassette ceiling unit	0.37	—	0.11	1.19	0.05	—	0.00	0.57	0.65	—	0.09	4.70
Portable unit exhaust pipe	4.98	—	0.87	28.62	9.35	—	1.06	82.67	1.28	—	0.05	34.88
Mechanical extract units	1.26	—	0.67	2.35	0.64	—	0.28	1.47	2.22	—	0.71	6.90
Freestanding fan	1.74	—	0.86	3.50	0.86	—	0.35	2.13	1.65	—	0.45	6.03
Unknown	1.15	—	0.46	2.89	1.91	—	0.58	6.33	0.72	—	0.14	3.76
Ventilation–bedroom												
Central air conditioning	Ref	.41			Ref	.10			Ref	.83		
Cassette ceiling unit	1.10	—	0.39	3.09	1.37	—	0.32	5.76	0.73	—	0.19	2.84
Mechanical extract units	1.86	—	0.98	3.56	2.28	—	1.03	5.05	1.27	—	0.49	3.26
Freestanding fan	1.29	—	0.43	3.88	0.38	—	0.09	1.64	1.34	—	0.28	6.50
Unknown	0.84	—	0.32	2.21	2.29	—	0.69	7.56	0.61	—	0.17	2.20
Subjective/time-varying factors												
Dining room temperature†‡	0.97	.73	0.81	1.16	1.00	.91	0.85	1.21	1.13	.36	0.87	1.48
Common room temperature†‡	0.96	.58	0.81	1.12	1.02	.83	0.84	1.24	0.96	.76	0.74	1.25
Bedroom temperature†‡	1.14	.25	0.91	1.43	1.19	.19	0.92	1.56	1.19	.09	0.98	1.45
Max people in common room†§	0.97	.15	0.94	1.01	—	—	—	—	0.99	.49	0.95	1.03
Low	—	—	—	—	Ref	.62			—	—	—	—
Medium	—	—	—	—	0.67		0.26	1.76	—	—	—	—
High	—	—	—	—	0.65		0.26	1.66	—	—	—	—
Max people in dining room†	0.99	.63	0.95	1.03	0.94	.032	0.89	0.99	1.00	.86	0.96	1.05
Washing floor frequency												
Less than daily	Ref				Ref				Ref			
Daily	1.63	.16	0.83	3.22	1.25	.64	0.49	3.17	2.38	.043	1.03	5.52
Air quality–common room												
Just right	Ref	.26			Ref	.69			Ref	.32		
Humid	0.61	—	0.34	1.10	0.74	—	0.33	1.64	0.59	—	0.29	1.18
Dry	0.76	—	0.36	1.62	1.08	—	0.39	2.94	0.79	—	0.32	1.93
Air quality–dining room												
Just right	Ref	.41			Ref	.98			Ref	.22		
Humid	0.77	—	0.44	1.34	0.92	—	0.44	1.94	0.73	—	0.39	1.38
Dry	1.40	—	0.56	3.49	0.95	—	0.28	3.22	1.84	—	0.66	5.14
Air quality–bedroom												
Just right	Ref	.86			Ref	.58			Ref	.61		
Humid	0.93	—	0.49	1.76	1.56	—	0.67	3.67	0.79	—	0.38	1.64
Dry	1.22	—	0.52	2.87	0.99	—	0.31	3.14	1.37	—	0.52	3.62
LA beds (%)	1.01	.59	0.99	1.01	1.00	.72	0.99	1.02	1.02	.024	1.00	1.03
Dementia beds (%)	1.00	.21	1.00	1.02	1.00	.37	0.99	1.01	1.01	.22	0.97	1.02

Models adjusted for variables in baseline models shown in Supplementary Table 4, Supplementary Table 5, Supplementary Table 6, Supplementary Table 7, interaction terms between Omicron period and prior immunity/vaccination variables retained in baseline models where statistically significant.

Models presented in Table 4 have frailty terms at individual and facility level.

∗ Adjusted for person-level: age, prior infection, receipt of second vaccine, sex; facility-level: Index of Multiple Deprivation, local SARS-CoV-2 incidence rate, for-profit status, number of beds, number of staff, number of residents, bed-to-resident ratio, resident-to-staff ratio, proportion residents with prior infection, proportion staff with prior infection, proportion staff vaccinated, proportion residents vaccinated.

† Median-centered.

‡ Per °Celsius increase. Temperatures >30 °C dropped from analysis.

§ Nonlinearly associated continuous variables presented as categorical variables in terciles.

In the stratified analysis pre-Omicron, an association was retained with more storeys (aIRR, 0.51; 95% CI, 0.28–0.94; *P* = .030), and bedrooms (1.04; 1.01–1.07; *P* = .006). Over this period, lower infection risk was associated with cassette ceiling unit ventilation compared with central air conditioning in the dining room (0.05; 0.00–0.57). Portable units with exhaust pipes increased risk more than 9-fold (9.35; 1.06–82.67), although wide CIs suggest uncertainty. In the Omicron-dominant period, purpose-built buildings retained the association with infection rate (2.92; 1.36–6.25; *P* = .006).

When considering time-varying or subjective variables that are more susceptible to bias, pre-Omicron, each additional person in the dining room reduced resident infection risk (0.94; 0.89–0.99; *P* = .032). During Omicron dominance, daily vs less frequent floor washing was associated with increased risk (2.38; 1.03–5.52; *P* = .043), as was a greater proportion of LA-funded beds (1.02; 1.00–1.03; *P* = .024) (Table 4).

Considering the second primary outcome of outbreak incidence (a measure of infection introduction), only community SARS-CoV-2 incidence affected risk in the “base” model (Supplementary Table 5). The aIRR for outbreak events comparing a high (75^th^ centile: 0.48 cases/100 population) vs low (25^th^ centile: 0.09 cases/100 population) local incidence was 2.84 (95% CI, 1.85–4.36; *P* < .001). Building factors had no associations with this outcome or interactions (Table 5).

**Table 5:** Mixed Effects Adjusted∗ Facility-level Models of Incidence of Outbreak (Poisson Model), Size of Outbreak (Negative Binomial Model), and Duration of Outbreak (Negative Binomial Model)

Building Factors	Incidence of Outbreaks				Outbreak Size				Outbreak Duration			
	aIRR	*P* Value	95% CI		aIRR	*P* Value	95% CI		aIRR	*P* Value	95% CI	
No. storeys	0.90	.37	0.71	1.14	0.91	.39	0.73	1.13	0.98	.83	0.78	1.22
Purpose built vs converted	1.10	.59	0.78	1.56	1.16	.36	0.84	1.57	0.90	.50	0.66	1.22
No. bedrooms†	1.00	.94	0.99	1.01	1.00	.31	1.00	1.02	1.00	.99	0.99	1.01
No. common rooms†	0.97	.34	0.91	1.03	1.00	.93	0.95	1.05	1.02	.47	0.97	1.08
No. dining rooms†	1.01	.85	0.89	1.15	1.00	.99	0.90	1.12	1.06	.29	0.95	1.19
Presence of shared bathrooms (staff with residents)	0.84	.31	0.60	1.18	0.93	.61	0.69	1.24	0.89	.47	0.66	1.21
Presence of shared bathrooms (between residents)	1.12	.74	0.56	2.24	0.76	.37	0.43	1.37	0.66	.18	0.35	1.22
Ventilation–common room												
Freestanding fan	Ref	.69			Ref	.13			Ref	.51		
Cassette ceiling unit	0.82	—	0.43	1.57	1.57	—	0.95	2.59	1.36	—	0.81	2.30
Portable unit exhaust pipe	1.58	—	0.54	4.69	1.53	—	0.66	3.54	1.54	—	0.67	3.50
Mechanical extract units	1.32	—	0.75	2.32	1.14	—	0.72	1.79	1.17	—	0.76	1.79
Central air conditioning	1.36	—	0.69	2.70	1.91	—	1.10	3.31	1.17	—	0.72	1.89
Unknown	1.06	—	0.52	2.18	1.41	—	0.81	2.46	1.62	—	0.92	2.84
Ventilation–dining room												
Central air conditioning	Ref	.72			Ref	.08			Ref	.05		
Cassette ceiling unit	0.72	—	0.16	3.32	1.25	—	0.55	2.83	0.70	—	0.26	1.87
Portable unit exhaust pipe	3.27	—	0.65	16.35	2.74	—	0.98	7.62	1.84	—	0.63	5.41
Mechanical extract units	0.83	—	0.45	1.50	1.00	—	0.67	1.48	1.17	—	0.77	1.79
Freestanding fan	0.97	—	0.50	1.86	1.30	—	0.84	2.02	1.27	—	0.79	2.06
Unknown	1.26	—	0.53	2.97	1.88	—	1.13	3.11	2.35	—	1.34	4.10
Ventilation–bedroom												
Central air conditioning	Ref	.72			Ref	.16			Ref	.79		
Cassette ceiling unit	0.82	—	0.27	2.49	2.04	—	0.85	4.89	1.40	—	0.57	3.44
Mechanical extract units	1.10	—	0.63	1.90	1.50	—	0.95	2.38	1.18	—	0.72	1.92
Freestanding fan	0.50	—	0.18	1.44	0.93	—	0.40	2.13	0.93	—	0.37	2.32
Unknown	1.00	—	0.45	2.21	1.52	—	0.84	2.75	1.35	—	0.72	2.52
Subjective/time-varying factors												
Dining room temperature†‡	1.06	.41	0.93	1.20	1.10	.11	0.98	1.23	1.00	.79	0.90	1.12
Common room temperature†‡	1.04	.46	0.93	1.17	1.05	.36	0.95	1.16	1.05	.34	0.95	1.17
Bedroom temperature†‡	1.11	.19	0.95	1.30	1.03	.61	0.91	1.17	1.15	.033	1.01	1.32
Max people in common room†	1.00	.69	0.98	1.01	0.99	.20	0.97	1.01	0.99	.16	0.97	1.00
Max people in dining room†	1.00	.75	0.98	1.02	1.00	.96	0.98	1.02	0.98	.009	0.96	0.99
Washing floor frequency												
Less than daily	Ref				Ref				Ref			
Daily	1.20	.34	0.82	1.76	1.20	.31	0.84	1.71	1.24	.25	0.86	1.77
Air quality–common room												
Just right	Ref	.75			Ref	.036			Ref	.94		
Humid	0.96	—	0.69	1.32	0.89	—	0.67	1.17	0.95	—	0.71	1.28
Dry	0.85	—	0.56	1.29	1.46	—	1.00	2.13	0.98	—	0.65	1.48
Air quality–dining room												
Just right	Ref	.94			Ref	.22			Ref	.96		
Humid	0.99	—	0.73	1.33	0.88	—	0.68	1.15	1.01	—	0.76	1.33
Dry	0.92	—	0.56	1.49	1.28	—	0.83	1.99	1.07	—	0.66	1.74
Air quality–bedroom												
Just right	Ref	.27			Ref	.53			Ref	.30		
Humid	1.31	—	0.93	1.84	0.88	—	0.66	1.18	1.08	—	0.80	1.47
Dry	0.97	—	0.61	1.54	1.12	—	0.73	1.71	0.74	—	0.47	1.17
LA beds (%)	1.00	.31	1.00	1.01	1.00	.36	0.99	1.00	0.99	.016	0.99	1.00
Dementia beds (%)	1.00	.74	1.00	1.01	1.00	.56	1.00	1.00	1.00	.68	1.00	1.00

Models in Table 5 include frailty term at care home level only.

∗ Adjusted for facility-level: median age in residents, proportion females among residents, Index of Multiple Deprivation, local SARS-CoV-2 incidence rate, for-profit status, number of beds, number of staff, number of residents, bed-to-resident ratio, resident-to-staff ratio, proportion residents with prior infection, proportion staff with prior infection, proportion staff vaccinated, proportion residents vaccinated.

† Median-centered.

‡ Per °Celsius increase. Temperatures >30°Celsius dropped from analysis.

For the overall analysis of outbreak size (reflecting transmission), outbreaks included 46% more cases in LTCFs with “dry” compared with “just right” common room air (aIRR, 1.46; 95% CI, 1.00–2.13) (Table 5), although this measure is highly subjective. There was one interaction with Omicron dominance: pre-Omicron, using portable units compared with central air conditioning in dining rooms increased outbreak size (7.29; 2.23–23.83).

**Fig. 2:**
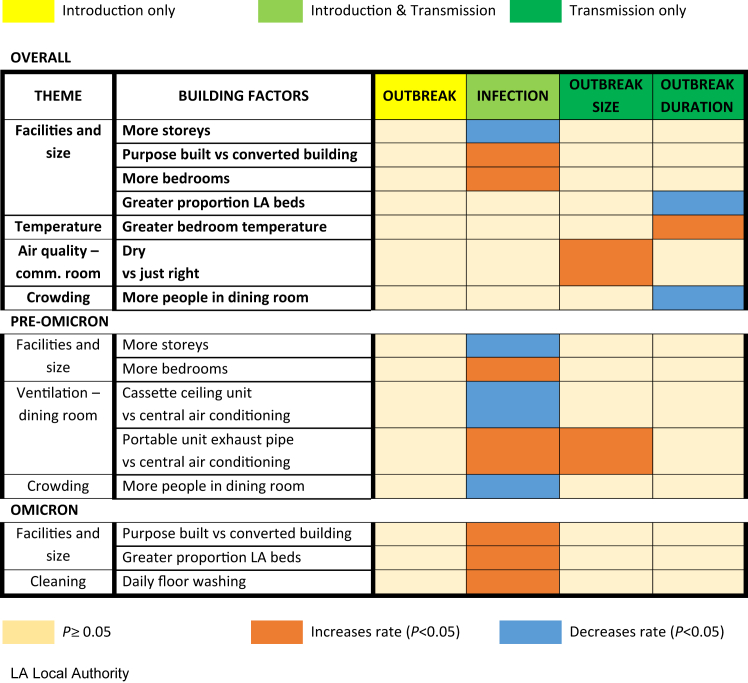
Heat map of building factors associated with outcomes overall and stratified into pre-Omicron period and Omicron-dominant period. Risk factors for outcomes describing introduction risk only are presented in the first column (outbreak), risk factors for transmission only are presented in the final 2 columns (outbreak size and outbreak duration), risk factors describing both introduction and transmission are presented in the second column (infection). Factors associated with increased risk of the outcome are shaded in orange and factors that are associated with a reduced outcome risk are shaded in blue. Results of overall analysis are shown in the top box, analyses stratified by Omicron period are presented in lower 2 boxes.

Outbreak duration was only associated with potentially time-varying covariates. A 1% reduction in duration was seen if LA funding increased by 1% (aIRR, 0.99; 95% CI, 0.99–1.00; *P* = .016) and by 2% with every extra person in the dining room (0.98; 0.96–0.99; *P* = .009). A 1°Celsius increase in bedroom temperature prolonged outbreaks by 15% (1.15; 1.01–1.32; *P* = .033). No interactions were identified (Table 5, Figure 2).

## Discussion

Our analysis demonstrated that the only clear driver of SARS-CoV-2 introduction into LTCFs was community incidence. However, building factors appeared to influence transmission within LTCFs, as they had important associations with outbreak characteristics and infection incidence in residents. Factors appearing to increase transmission included purpose-build, more bedrooms, and warmer temperatures. Transmission appeared lower in LTCFs with more storeys, and ceiling-mounted compared with central air conditioning. Ventilation type also affected transmission in the pre-Omicron period. These factors are mainly indicators of airflow and how well LTCFs can isolate infected residents,^5^^,^^31^ for example by caring for them on different floors. Limiting spread may therefore be more achievable for LTCFs than stopping infection introduction. Subjective and time-varying factors associated with increased transmission included drier perceived air, and frequent cleaning (during Omicron dominance only). Conversely, reduced transmission was seen in LTCFs withs more LA-funded beds and more people in common spaces.^5^^,^^30^ These relationships may reflect underlying confounding or reverse causality. Nevertheless, we found substantial diversity in built environments, highlighting that local expertise can optimize infection control strategies.

Factors available from administrative datasets known to influence SARS-CoV-2 outcomes include staffing, occupancy, for-profit status, rurality, and community incidence.^10^^,^^32^, ^33^, ^34^, ^35^ However, data are scant regarding the heterogeneity in LTCF built environments in relation to infection control. Most LTCFs reported older central air conditioning or freestanding fans and although confidence intervals were wide, ventilation was associated with transmission risk (reduced risk with ceiling-mounted units and greater risk with portable units). This may relate to whether systems recirculate cooled air or draw in outdoor air.^36^^,^^37^ Although we did not specifically ask about filters, this will be addressed by the recently funded AFRI-c study.^38^ Even using newer ventilation systems, strategies such as CO_2_ monitoring (proxy for overcrowding), which triggers air refreshment, may not be suitable for LTCFs^39^ given specific characteristics of this population (eg, reduced mobility) and may need recalibration. To date, no large studies in LTCFs have evaluated how ventilation affects infection spread.^40^

After adjustment, purpose-built buildings had almost twofold greater rate ratio for infection than converted ones, which is surprising. New LTCF building standards were introduced in 2003^41^ but, of 34 responses, 24 facilities were built pre-2003 and were not subject to the updated guidance. It is also possible that air leakage from external envelopes of older converted homes may reduce transmission. Apparent increased transmission in warmer environments may have been affected by measurement bias. Consistent with published literature,^9^^,^^42^ drier air was associated with lower transmission, although assessments were subjective and the complex interplay among temperature, humidity, and airflow precludes meaningful conclusions.^9^^,^^31^^,^^42^

We present a comprehensive description of LTCF built environments in a diverse sample of facilities. The sample is broadly generalizable to the LTCF population in England in view of its geographic distribution and provider representation. However, compared with the national average, a greater proportion of our sample was for-profit (87% vs 82%) and LTCFs were larger (average 53 vs 31 beds),^43^^,^^44^ which is more similar to LTCFs in the United States, Italy, Germany, and Spain, where facilities are larger, although for-profit ownership is less prevalent in European countries.^3^^,^^45^ Our study considered outcomes describing both introduction and transmission, generating more readily applicable evidence for policy. We explored this during the pandemic peak, which provided a unique opportunity to monitor infection in LTCFs because of regular asymptomatic COVID-19 testing across the care sector. In contrast to published studies accessing aggregate data, we linked test results from individuals to specific LTCFs and estimated entry and exit dates, which is particularly important given the high turnover.^46^ Nineteen-month follow-up allowed us to consider how emerging variants affected associations.

This study was limited by missing data, mostly affecting questions with less readily accessible answers. Questionnaires were distributed during a significantly strained period for the sector, and they were therefore completed after the study start. As such, reverse causality may have affected results, for example LTCFs with larger outbreaks probably subsequently cleaned more frequently. Non-response bias is possible as more severely affected LTCFs may not have responded, although we achieved a response rate of 91%. Many variables were subjective and social desirability bias is possible as answers may have reflected best practice. Simultaneous policy changes were difficult to account for, although models were adjusted for calendar month and variation in local incidence, population characteristics, and immunity. Inferences around certain associations were imperfect; for example, temporal changes in indoor temperature and air quality were not captured cross-sectionally, and unmeasured factors such as policies around discharge from hospitals into LTCFs probably affected LA funding of beds. As multiple variables have been considered, significant associations may have been detected by chance.

## Conclusions and Implications

We have comprehensively described diversity in LTCF built environments and highlighted associations with infection transmission in LTCFs. Research considering these relationships should inform preventive policy and guidelines. Limiting infection spread is probably more achievable than preventing introduction, and characteristics such as outbreak size and duration may help identify LTCFs that would benefit from targeted support. Based on our findings, LTCFs that may be better at preventing infection spread have fewer bedrooms, better ventilation, cooler air, and facilities to cohort infected residents, for example on different floors. Some of these features are reflected in the Green House model, where residents live in small, self-contained units with designated staff. Pre-pandemic studies found improved quality of life and lower hospital admission rates among residents,^47^ who also experienced lower COVID-19 incidence and mortality in the first pandemic wave.^48^ UK LTCF standards were last updated 20 years ago, and new standards should build on momentum gained in the pandemic to optimize preventive approaches against future respiratory infectious threats while facilitating well-being and dignity for residents.

### Data Availability

De-identified test results and limited metadata will be made available for use by researchers in future studies, subject to appropriate research ethical approvals once the VIVALDI study cohort has been finalized. These datasets will be accessible via the Health Data Research UK Gateway (https://www.hdruk.ac.uk/).

## Figures and Tables

**Table:** Baseline data

Role of Interviewee			Survey Date		
Care Home CQC-ID Number			No. of Residents		
Location, town City					
No. member of staff	Night shift		No. of visits in average	weekdays	
	Day shift			weekends	
Dates when home closed to visitors since 1 March 2020					

**Table:** Building typology (Tick all that apply)

Type	Converted	Purpose Built	No Floors	Year of Construction (if known)	No. of Bedrooms or Apartments	
					Bedrooms	Apartments
House						

**Table:** How many communal areas are there in the care home?

Room Type	Number	Others Communal Areas (Please Indicate Below)	Number	Others Communal Areas (Please Indicate Below)	Number
Common room/s					
Dining room/s					
Kitchen					
shared toilets					
Corridors					
staircase					

**Table:** What types of heating system/s does the care home have and use? (Tick all that apply)

Heating Systems	Communal Areas							Bedrooms or Apartments	
	Common Room/s	Dining Room/s	Shared Toilets	Others (Please Indicate Below)					
								Bedrooms	Apartments
Central heating (radiators, warm-air heater or under-floor heating)									
Portable heater (electric, oil-filled, bottled gas, paraffin)									
Other (please state)									
Not heated									
Don't know									
If central heating, indicate the setting temperature (^°^C)?									

**Table:** What types of mechanical ventilation system does the care home use? (Tick all that apply)

Ventilation Systems	Communal Areas							Bedrooms or Apartments	
	Common Room/s	Dining Room/s	Shared Toilets	Others (Please Indicate Below)					
								Bedrooms	Apartments
Central air conditioning system									
Cassette ceiling units									
Portable units exhaust pipes									
MVHR (Mechanical ventilation with heat recovery)									
Mechanical extract units									
Freestanding fans									
Other (please state)									
Don't know									
Do you know if any of the rooms has humidification or air purifiers?									
Do you know if the MVHR has a *thermal wheel*?									

**Table:** What is the frequency of opening windows to outside air during weekdays? (Tick all that apply)

Opening Windows Weekdays	Communal Areas							Bedrooms or Apartments	
	Common Room/s	Dining Room/s	Shared Toilets	Others (Please Indicate Below)					
								Bedrooms	Apartments
Every day, all windows									
Every day, some windows									
Once per week, all windows									
Once per week, some windows									
Rarely									
Do not open									
If not, why?									

**Table:** What is the frequency of opening windows to outside air during weekends? (Tick all that apply)

Opening Windows Weekends	Communal Areas							Bedrooms or Apartments	
	Common Room/s	Dining Room/s	Shared Toilets	Others (Please Indicate Below)					
								Bedrooms	Apartments
Both days, all windows									
Both days, some windows									
Once or twice per month, all windows									
Once or twice per month, some windows									
Rarely									
Do not open									
If not, why?									

**Table:** In the chart below, please indicate mealtimes and times of any planned activities in the care home along with the area that these usually take place in.

Activity	Weekday Times (eg, 7–9)	Weekend Times	Activity Held in (eg, Dining Room)
Breakfast			
Lunch			
Tea			
Dinner			
Planned activity (please state)			
Planned activity (please state)			
Planned activity (please state)			

**Table:** Please in the chart below, indicate the number of people using the communal areas during a typical weekday. Write the average number of people as shown in the example below.

Example (Time of day) (24-h Clock)		Before 5	Breakfast				10	11	Lunch			15	16	17	Dinner			After 22
			6	7	8	9			12	13	14				18	19	20	
Common rooms	1			10			5			20			5			12		

		before 5	breakfast						lunch						dinner			
			6	7	8	9	10	11	12	13	14	15	16	17	18	19	20	After 22
Common rooms	1																	
	2																	
	3																	
	4																	

		before 5	breakfast						lunch						dinner			
			6	7	8	9	10	11	12	13	14	15	16	17	18	19	20	After 22
Dining rooms	1																	
	2																	
	3																	
	4																	

		before 5	breakfast						lunch						dinner			
			6	7	8	9	10	11	12	13	14	15	16	17	18	19	20	After 22
Other common rooms	1																	
	2																	
	3																	
	4																	

**Table:** What is the frequency of ……. common areas? (Tick all that apply)

	Vacuuming Floor	Washing Floor	Sweeping Floor
Everyday			
Once a week			
Few times a week			
Few times a month			
Once in a month			

**Table:** How would you describe the quality of the air in the care home?

	Too Humid	Humid	Slightly Humid	Just Right	Slightly Dry	Dry	Too Dry
Common room/s							
Dining room/s							
Kitchen area							
Shared toilets							
Bedrooms or apartments							

**Supplementary Table 1:** Covariates Included in Baseline Model

Covariate	Infection Incidence in Resident	Outbreak Incidence	Outbreak Size	Outbreak Duration
Individual-level				
Sex	X			
Age	X			
2^nd^ vaccine dose∗	X			
Prior infection∗	X			
Facility-level				
Proportion >80 years∗		X	X	X
Proportion residents female∗		X	X	X
Proportion with prior infection (res)∗	X	X	X	X
Proportion with prior infection (staff)∗	X	X	X	X
Proportion fully vaccinated (res)∗	X	X	X	X
Proportion fully vaccinated (staff)∗	X	X	X	X
Number of residents∗	X	X	X	X
Number of staff∗	X	X	X	X
Number of beds	X	X	X	X
Staff-to-resident ratio∗	X	X	X	X
Bed-to-resident ratio∗	X	X	X	X
Local infection incidence∗	X	X	X	X
IMD decile	X	X	X	X
Analysis month	X	X	X	X

∗ Time-varying.

**Supplementary Table 2:** Mean Indoor Temperatures and Number of Responses Reported by Month of Survey Completion in Dining Room, Common Room, and Bedroom

Month of Completion	Dining Room (°C) (SD)	No. Responses	Common Room (°C) (SD)	No. Responses	Bedroom (°C) (SD)	No. Responses
April	22.6 (2.0)	14	23 (2.0)	18	22 (2.0)	12
May	20.3 (1.5)	3	20.8 (1.5)	4	19.5 (0.7)	2
June	21 (0)	1	23 (2.8)	2	—	0
July	22 (1)	3	21.5 (1.0)	6	22 (1)	3
August	26 (2.8)	2	25 (2.6)	3	26 (2.8)	2
September	22.9 (3.5)	7	23.3 (3.4)	7	23.5 (4.7)	4
October	23 (1)	3	22 (0.8)	4	22 (0.8)	4
November	—	0	25 (0)	1	—	0

In line with main analysis, values >30 °C were treated as nulls.

**Supplementary Table 3:** Characteristics of Outbreaks According to Dominant Variant Type

Dominant Variant	Wild-Type	Alpha	Delta	Omicron
Outbreak Characteristics (Mean, SD)				
Number of outbreaks	63	57	49	144
Outbreak size	23.4 (27.5)	15.4 (14.5)	15.6 (25.2)	31.2 (24.1)
Residents	12.5 (14.2)	7.8 (7.6)	6.9 (10.2)	12.4 (10.0)
Staff	9.9 (14.1)	6.6 (7.9)	7.7 (15.5)	17.8 (15.1)
Outbreak duration, d	29.2 (25.4)	18.7 (14.7)	31.7 (36.3)	57.4 (42.1)
Outbreaks per LTCF	1.07 (0.25)	1.03 (0.19)	1.07 (0.25)	1.17 (0.38)

Wild-type interval September 1, 2020–December 31, 2020; Alpha interval January 1, 2021–May 31, 2021; Delta interval June 1, 2021–November 30, 2021; Omicron interval December 1, 2021–March 31, 2022.

**Supplementary Table 4:** Mixed Effects Multivariate Poisson Regression Analysis for Incidence of SARS-CoV-2 Infection in LTCF Residents Over the Study Period

Covariates and Covariate Categories	Unstratified, n = 88,057				Stratified – Pre-Omicron, n = 68,185				Stratified – Omicron, n = 19,872 ′			
	aIRR	*P* Value	95% CI		aIRR	*P* Value	95% CI		aIRR	*P* Value	95% CI	
Individual-level												
Age∗	1.02	<.001	1.01	1.02	1.01	.001	1.00	1.02	1.02	<.001	1.02	1.03
Age squared	1.00	<.001	1.00	1.00	1.00	.005	1.00	1.00	1.00	<.001	1.00	1.00
Female vs male	1.01	.77	0.93	1.10	0.97	.60	0.85	1.10	1.05	.46	0.93	1.18
Receipt of 2nd vaccine dose	0.66	<.001	0.53	0.82	0.53	.002	0.36	0.80	0.66	.002	0.51	0.85
Prior infection					0.19	<.001	0.13	0.30	0.70	.002	0.56	0.88
LTCF level												
Local SARS-CoV-2 IR	50.83	<.001	28.84	89.58	204,979.20	<.001	26,990.93	1,556,689.00	21.98	<.001	5.53	87.36
Local IR squared	0.32	<.001	0.24	0.42	0.00	<.001	0.00	0.00	0.46	.010	0.26	0.83
IMD decile		.74				.82				.64		
1	Ref				Ref				Ref			
2	0.97		0.38	2.49	0.77		0.23	2.60	1.08		0.34	3.37
3	1.32		0.52	3.37	1.16		0.34	4.00	1.57		0.50	4.94
4	0.99		0.35	2.75	1.96		0.52	7.33	0.62		0.18	2.18
5	0.94		0.39	2.27	1.01		0.32	3.20	0.97		0.33	2.86
6	0.82		0.33	2.04	0.75		0.23	2.47	0.91		0.30	2.76
7	0.73		0.27	1.97	0.64		0.18	2.33	0.68		0.21	2.23
8	0.70		0.27	1.81	0.79		0.23	2.71	0.54		0.17	1.69
9	0.47		0.16	1.37	0.55		0.14	2.22	0.52		0.14	1.87
10	0.55		0.21	1.45	0.56		0.16	2.01	0.45		0.13	1.50
For-profit vs not-for-profit	1.69	.12	0.87	3.31	0.90	.82	0.38	2.15	1.54	.31	0.67	3.54
Total beds∗	1.00	.63	0.99	1.01	1.00	.80	0.99	1.02	1.01	.18	0.99	1.03
Bed-to-resident ratio∗	7.18	<.001	4.52	11.40	13.90	<.001	7.38	26.19	1.02	.97	0.37	2.80
Bed-to-resident ratio squared	1.78	<.001	1.44	2.21	2.17	<.001	1.73	2.70				
Resident-to-staff ratio∗	0.31	<.001	0.22	0.43					1.83	.28	0.60	5.57
No. residents∗	1.01	.20	1.00	1.01	1.02	.005	1.00	1.03	1.00	.67	0.98	1.03
No. residents squared	1.00	.019	1.00	1.00					1.00	.04	1.00	1.00
No. staff∗	0.99	<.001	0.99	1.00	0.97	<.001	0.97	0.98	0.99	.04	0.98	1.00
No. staff squared	1.00	.07	1.00	1.00	1.00	<.001	1.00	1.00				
Fully vaccinated residents (%)	0.99	.11	0.98	1.00	1.00	.82	0.98	1.01	4.80	.02	1.25	18.39
Fully vaccinated residents squared									0.99	.02	0.98	1.00
Fully vaccinated staff (%)					0.98	<.001	0.97	0.99	0.97	.10	0.93	1.01
Prior infection in staff (%)					0.93	.008	0.97	0.99	1.00	.94	0.99	1.01
Prior infection in residents (%)					0.96	<.001	0.95	0.97	0.92	<.001	0.91	0.93
Interactions												
Omicron & fully vaccinated staff in LTCF (%)												
Pre-Omicron	1.00	.55	0.99	1.02								
Omicron	0.74	.25	0.44	1.24								
Omicron & fully vaccinated staff in LTCF (%) squared												
Pre-Omicron	1.00	.07	1.00	1.00								
Omicron	1.00	.37	1.00	1.00								
Omicron & individual-level prior infection status												
No prior infection	Ref											
Pre-Omicron	0.18	<.001	0.12	0.28								
Omicron	0.69	.001	0.55	0.87								
Omicron & prior infection in residents in LTCF (%)												
Pre-Omicron	0.97	<.001	0.95	0.98								
Omicron	0.89	<.001	0.88	0.91								
Omicron & prior infection in residents in LTCF (%) squared												
Pre-Omicron	1.00	.92	1.00	1.00								
Omicron	1.00	<.001	1.00	1.00								
Omicron & prior infection in staff in LTCF (%)												
Pre-Omicron	1.00	.68	0.98	1.01								
Omicron	1.00	.57	0.99	1.01								

IMD, Index Multiple Deprivation.

Two frailty terms: at LTCF level and at individual level.

Interactions with *P* value < .05 presented.

∗ Median-centered no. temperatures >30 °C dropped from analysis.

**Supplementary Table 5:** Mixed Effects Multivariate Poisson Regression Analysis for Incidence of SARS-CoV-2 Outbreak in an LTCF Over the Study Period

Covariates and Covariate Categories	aIRR	*P* Value	95% CI		Interaction∗*P* Value
Median age in residents†	1.00	.67	0.97	1.05	
Proportion females among residents (%)	1.01	.10	1.00	1.02	
Local IR	28.25	<.001	6.40	124.58	
Local IR squared	0.31	.003	0.14	0.68	
IMD decile		.92			
1	Reference				
2	1.01		0.63	1.63	
3	1.04		0.64	1.68	
4	0.93		0.55	1.57	
5	0.88		0.55	1.39	
6	0.88		0.55	1.42	
7	0.76		0.45	1.29	
8	0.75		0.45	1.24	
9	0.83		0.47	1.45	
10	0.72		0.43	1.23	
For-profit vs not-for-profit	1.02	.93	0.70	1.47	
Bed-to-resident ratio†	0.99	.98	0.58	1.69	
Resident-to-staff ratio†	0.74	.28	0.42	1.28	
Total beds†	1.00	.79	0.99	1.01	
Number of staff in LTCF†	1.00	.90	0.99	1.01	
Number of residents in LTCF†	1.01	.08	1.00	1.02	
Residents with 2nd vaccine in LTCF (%)	0.97	.06	0.94	1.00	.69
Staff with 2nd vaccine in LTCF (%)	1.00	.85	0.99	1.02	.69
Prior infection in residents (%)	1.00	.31	0.98	1.01	.23
Prior infection in staff (%)	0.98	.05	0.97	1.00	.95

IMD, Index Multiple Deprivation.

Frailty term at LTCF level.

∗ Interaction with Omicron period.

† Median-centered no. temperatures >30 °C dropped from analysis.

**Supplementary Table 6:** Mixed Effects Multivariate Negative Binomial Regression Analysis for Size of SARS-CoV-2 Outbreak in an LTCF Over the Study Period

Covariates and Covariate Categories	aIRR	*P* Value	95% CI		Interaction∗*P* Value
Median age in residents†	1.00	.99	0.96	1.04	
Proportion females among residents (%)	0.99	.07	0.98	1.00	
Local IR	1.35	.26	0.80	2.27	
IMD decile		.029			
1	Reference				
2	1.00		0.66	1.53	
3	1.05		0.68	1.62	
4	1.11		0.70	1.75	
5	0.89		0.58	1.36	
6	0.76		0.50	1.15	
7	0.69		0.44	1.08	
8	0.70		0.45	1.11	
9	1.18		0.71	1.96	
10	0.52		0.32	0.83	
For-profit vs not-for-profit	1.00	.98	0.72	1.38	
Total beds†	1.00	.38	0.99	1.00	
Bed-to-resident ratio†	2.09	.12	0.83	5.23	
Bed-to-resident ratio squared	4.13	.040	1.07	16.04	
Resident-to-staff ratio†	2.32	.025	1.11	4.83	
Resident-to-staff ratio squared	0.27	.014	0.09	0.76	
No. residents in LTCF∗†	1.01	.038	1.00	1.02	
No. staff in LTCF†	1.01	.001	1.00	1.02	
Residents with 2nd vaccine in LTCF (%)	1.00	.68	0.98	1.03	.29
Staff with 2nd vaccine in LTCF (%)	0.92	<.001	0.89	0.96	.47
Staff with 2nd vaccine in LTCF (%) squared	1.00	<.001	1.00	1.00	
Prior infection in staff (%)	1.00	.90	0.99	1.02	.33
Interactions					
Omicron & proportion of residents with prior infection (%)					.002
Pre-Omicron	0.91	<.001	0.88	0.94	
Omicron	0.98	.033	0.96	1.00	
Omicron & Proportion of residents with prior infection (%) squared					N/A
Pre-Omicron	1.00	<.001	1.00	1.00	
Omicron	1.00	.17	1.00	1.00	

IMD, Index Multiple Deprivation.

Frailty term at LTCF level.

∗ Interaction with Omicron period.

† Median-centered no. temperatures >30 °C dropped from analysis.

**Supplementary Table 7:** Mixed Effects Multivariate Negative Binomial Regression Analysis for Duration of SARS-CoV-2 Outbreak in an LTCF Over the Study Period

Covariates and Covariate Categories	aIRR	*P* Value	95% CI		Interaction∗*P* Value
Median age in residents†	1.00	.92	0.96	1.03	
Proportion females among residents (%)	1.00	.48	0.98	1.01	
Local IR	1.04	.90	0.60	1.79	
IMD decile		.82			
1	Reference				
2	0.78		0.51	1.21	
3	0.75		0.47	1.18	
4	0.92		0.58	1.48	
5	0.71		0.46	1.09	
6	0.79		0.51	1.22	
7	0.70		0.44	1.12	
8	0.74		0.47	1.18	
9	0.75		0.45	1.26	
10	0.63		0.39	1.01	
For-profit vs not-for-profit	0.87	.40	0.63	1.20	
Bed-to-resident ratio†	1.07	.80	0.62	1.86	
Resident-to-staff ratio†	2.01	.06	0.96	4.18	
Resident-to-staff ratio squared	0.33	.05	0.11	1.02	
Total beds†	1.00	.28	0.99	1.00	
Number of residents in LTCF†	1.01	.36	0.99	1.02	
Number of staff in LTCF†	1.01	<.001	1.01	1.02	
Number of staff in LTCF squared	1.00	.014	1.00	1.00	
Residents with 2nd vaccine in LTCF (%)	1.03	.022	1.00	1.06	.45
Staff with 2^nd^ vaccine in LTCF (%)	0.99	.33	0.97	1.01	.22
Prior infection in residents (%)	0.99	.13	0.98	1.00	.63
Prior infection in staff (%)	1.00	.83	0.99	1.02	.38

IMD, Index of Multiple Deprivation.

Frailty term at LTCF level.

∗ Interaction with Omicron period.

† Median-centered no. temperatures >30 °C dropped from analysis.

## Supplementary Material 1. Supplementary Methods

### Data Linkage

As previously described,^49^^,^^50^ results of LFD/PCR tests were used to link results from individual residents and staff to specific LTCFs using CQC-ID, a unique number allocated by the Care Quality Commission to each care facility. Using a person-level pseudo-identifier based on National Health Service number, results were linked to vaccination records held in the National Immunisations Management System and to anti-nucleocapsid SARS-CoV-2 antibody test results from a subset of consenting VIVALDI participants. Individual-level data linkage was undertaken within the COVID-19 datastore, a secure online repository commissioned by NHS England.^51^ LTCF size was also retrieved from the Capacity Tracker dataset, a regularly self-completed tool documenting LTCF capacity.^21^ Using LTCF address, linkage was performed to local SARS-CoV-2 incidence,^26^ and to the Index for Multiple Deprivation.^25^ The number of beds funded by the local authority or for dementia care was obtained directly from the LTCFs.

### Variable Selection–Baseline Model

Individual-level covariates were age (centered at the median for analysis), sex, prior infection (any prior positive LFD/PCR or antibody test), vaccination status (≥2 vs <2 vaccinations more than 14 days prior). Anti-nucleocapsid-antibody was tested using the Abbott ARCHITECT i-system immunoassay; positivity threshold was 0.8 IU.^20^ Facility-level covariates were median-centered for analysis, including number of beds, bed-to-resident ratio, resident-to-staff ratio, number of staff, and number of residents. Other facility-level covariates included the proportion of those who had received at least 2 vaccinations, had prior infection, and/or were female (residents). We also included the local SARS-CoV-2 incidence rate, Index of Multiple Deprivation (IMD) decile, and for-profit status. Where a monthly record was unavailable, the closest preceding entry was used.

**2. Built environment survey**

Do you know if the windows in the care home have restrictors that limit their openings?

No, they don't have restrictors.

Yes, all of them.

Yes, some of them (mainly in bedrooms)

Yes, some of them (mainly in communal areas)

Do any of the doors in the communal areas open to the outside?

No.

Yes.

If yes, where: …………………………………………………………………………………………………………………………………

how many: …………………………… how long they kept open? ……………………………………………………………

Are there double bedrooms shared by residents?

No.

Yes.

If yes, how many double bedrooms?......................................................................................................

Are there communal (shared) toilets or bathrooms?

No.

Yes.

If yes, how many?.....................................................................................................................................

Do residents share toilets with staff and/or visitors?

No.

Yes.

If yes, where they are located?................................................................................................................

Do you ever have condensation in any room of the care home?

No.

Yes.

If yes, in which rooms does condensation occur?...................................................................................

Are there water dispensers or water fountains in communal areas?

No.

Yes.

If yes, how many and where?..................................................................................................................

Are there tea and coffee points in communal areas?

No.

Yes.

If yes, where?..........................................................................................................................................

Is there a garden or outside space that residents have access and use during the day?

No.

Yes.

If yes, how many people use it on average and for how long?...................................................................
